# GMF-Net: A Gaussian-Matched Fusion Network for Weak Small Object Detection in Satellite Laser Ranging Imagery

**DOI:** 10.3390/s26020407

**Published:** 2026-01-08

**Authors:** Wei Zhu, Weiming Gong, Yong Wang, Yi Zhang, Jinlong Hu

**Affiliations:** 1 Institute of Seismology, China Earthquake Administration, Wuhan 430071, Chinazhangyi246@mails.ucas.ac.cn (Y.Z.); 2 Hubei Key Laboratory of Earthquake Early Warning, Hubei Earthquake Agency, Wuhan 430071, China; 3 Xinjiang Astronomical Observatory, Chinese Academy of Sciences, Urumqi 830011, China; wangyong@xao.ac.cn

**Keywords:** satellite laser ranging (SLR), small object detection, lightweight network, GMF-Net, Gaussian-matched convolution

## Abstract

Detecting small objects in Satellite Laser Ranging (SLR) CCD images is critical yet challenging due to low signal-to-noise ratios and complex backgrounds. Existing frameworks often suffer from high computational costs and insufficient feature extraction capabilities for such tiny targets. To address these issues, we propose the Gaussian-Matched Fusion Network (GMF-Net), a lightweight and high-precision detector tailored for SLR scenarios. The core scientific innovation lies in the Gaussian-Matched Convolution (GMConv) module. Unlike standard convolutions, GMConv is theoretically grounded in the physical Gaussian energy distribution of SLR targets. It employs multi-directional heterogeneous sampling to precisely match target energy decay, enhancing central feature response while suppressing background noise. Additionally, we incorporate a Cross-Stage Partial Pyramidal Convolution (CSPPC) to reduce parameter redundancy and a Cross-Feature Attention (CFA) module to bridge multi-scale features. To validate the method, we constructed the first dedicated SLR-CCD dataset. Experimental results show that GMF-Net achieves an mAP@50 of 93.1% and mAP@50–95 of 52.4%. Compared to baseline models, parameters are reduced by 26.6% (to 2.2 M) with a 27.4% reduction in computational load, demonstrating a superior balance between accuracy and efficiency for automated SLR systems.

## 1. Introduction

Satellite Laser Ranging (SLR) has become one of the most accurate technologies for space target ranging, based on the high brightness, monochromaticity, high directionality, and strong coherence of laser light. By precisely determining the round-trip propagation time delay of laser pulses between the observatory and the target satellite and by combining it with the speed of light to solve the distance parameter, SLR has become one of the most accurate technologies for space target ranging [1,2,3,4]. With its high precision and strong anti-interference capability, it has been successfully applied to the study of Earth rotation dynamics [5], satellite precision orbiting [6,7], time–frequency transfer [8], gravitational wave detection [9], climate monitoring [10], space debris monitoring [11], and other fields. In modern SLR systems, charge-coupled devices (CCDs), as the core component of the telescope’s optoelectronic sensing unit, are capable of acquiring high-resolution images in real time to provide visualization support for automated tracking. Although the automated control system has greatly improved observing efficiency, manual fine-tuning using CCD images is still required to compensate for target offset caused by atmospheric disturbances, optical axis deviations, and ephemeris errors. Especially under low-light observation conditions at night, limited by weak reflected signals and complex background noise (such as moonlight interference, dark current noise, and readout noise), the signal-to-noise ratio (SNR) of the CCD image is greatly reduced. Consequently, the system faces the challenges of weak signal extraction under low-light conditions and small target detection in a noisy background (with a typical target size of only about 8×8 pixels). The system faces key technical challenges such as low illumination, weak signal extraction, and small target detection in a noisy background.

### 1.1. Technical Bottlenecks in Target Detection in SLR-CCD Images

Although numerous studies have attempted to apply object detection methods to SLR scenarios, significant challenges remain in the following key areas:

1. **Inadequate feature modeling and target adaptability**:
  Objects in SLR images typically follow an approximate Gaussian distribution, with energy highly concentrated in the central region and decaying rapidly toward the periphery (see Section 2.2 for data analysis). Traditional convolutional structures—with fixed receptive fields and no directional sensitivity—struggle to extract these spatial features effectively. Moreover, when standard convolutional kernels shift across a uniform grid, they assign equal weight to low-energy peripheral regions and high-energy central regions, which increases noise interference and dilutes the true signal.
2. **Mismatch between algorithms and practical applications**:
  Traditional detection methods rely on manually designed features (e.g., Haar wavelet [12], Viola-Jones integral map [13], HOG features [14], etc.), which lack robustness in complex backgrounds. In deep learning, two-stage detectors (e.g., the R-CNN series [15,16,17]) achieve high accuracy but incur high computational complexity due to their multi-stage structure, making them unsuitable for real-time deployment. Single-stage detectors (e.g.SSD [18], YOLO series [19,20,21,22,23,24]) have become the mainstream solution for real-time inference and have been applied in drone imagery [25], infrared detection [26], satellite-component detection [27], landslide monitoring [28], and fruit classification [29]. However, most still rely on generic feature modeling and have not incorporated the dual characteristics of SLR targets—Gaussian energy distribution and extremely small scale—for structural optimization, resulting in suboptimal performance.
3. **Overly simple and redundant feature fusion strategies**:
  Current networks such as YOLO suffer from limited inter-layer interaction and channel utilization. Although fusion modules like FPN, PANet, and CSP/C2f have been introduced, they typically merge multi-scale features via simple concatenation or static weighting, without dynamic adaptation to differing energy distributions. This makes it difficult to jointly enhance small-target distinguishability. Furthermore, the abundance of redundant channels and convolutional operations—while enriching representation—substantially increases model size and computational overhead, hindering lightweight deployment.

In summary, effective SLR small-target detection demands systematic optimization in three aspects—feature modeling, algorithm design, and feature fusion—to fully exploit the Gaussian-distributed, tiny nature of satellite laser returns.

### 1.2. Innovations and Contributions of This Paper

In order to address the aforementioned challenges, this paper proposes a lightweight, high-precision real-time detection network—GMF-Net (Gaussian-Matched Fusion Network)—whose key innovations include the following:

1. **Gaussian-Matched Convolution (GMConv):** Convolution kernels of a traditional rectangular nature, characterized by uniform sensing, are inadequate in accurately matching the approximate Gaussian distribution characteristics of SLR targets. These targets are distinguished by “central energy concentration and rapid outward decay,” as analyzed in Section 2.2. To address this issue, the GMConv module was developed. This module employs a synergistic mechanism of multi-directional heterogeneous sampling and dynamic channel calibration to adaptively capture Gaussian energy distribution features, enhancing central response while effectively suppressing background noise.
2. **Cross-Stage Partial Pyramid Convolution (CSPPC):** In order to achieve model lightweighting, this paper introduces partial convolution (Partial Conv) mechanisms and a pyramid structure into the neck network, constructing the CSPPC module. This design effectively reduces model parameters and computational complexity while enhancing the network’s multi-scale fusion capabilities.
3. **Cross-Feature Attention (CFA):** The CFA module has been developed to address the limitations of traditional detectors in making independent predictions at each level and severing cross-scale feature correlations. The module integrates shallow-layer, high-resolution details with deep-layer semantic information through cross-layer feature map fusion and local attention mechanisms, significantly enhancing multi-scale object detection capabilities.
4. **The construction of the SLR-CCD Dataset:** Addressing the paucity of data in this domain, this paper establishes the first SLR-CCD dataset covering complex noise and multi-scale features, providing a robust data foundation for model training and algorithm validation.

## 2. Dataset Construction and Objective Analysis

### 2.1. Dataset Construction

The optimization of deep learning model performance is contingent upon the availability of high-quality training data. This study was conducted in response to the paucity of charge-coupled device (CCD) image datasets in the Satellite Laser Ranging (SLR) field. The objective of this study was to collect data within the operational environment of the TROS1000 system. TROS1000, developed by the Institute of Seismology, China Earthquake Administration, is the world’s largest-aperture mobile SLR system. The system is equipped with a one-meter optical telescope, with a maximum ranging distance of 36,000 km. It is deployed at the Nanshan Station of the Xinjiang Astronomical Observatory, Chinese Academy of Sciences. The collected data encompass a range of atmospheric conditions and noise levels, from which 2000 frames were selected for annotation.

In order to enhance the model’s generalization capability and multi-task extensibility, this study employed the DarkLabel 2.4 professional annotation tool to simultaneously label SLR targets and non-ranging moving objects (e.g., space debris) within the field of view. This annotation strategy facilitates the accurate screening of candidate target regions during SLR observations and provides data support for multi-task sky-background detection.

**Figure 1:**
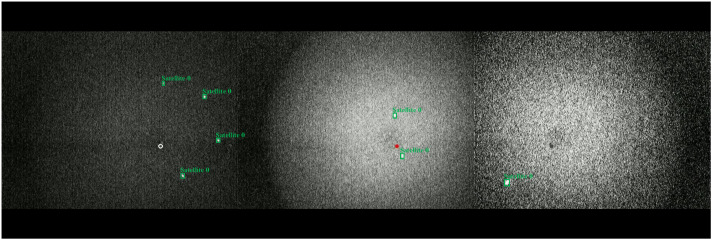
Examples from the SLR-CCD dataset. (**Left**) Samples collected under low-illumination, high-noise conditions. (**Middle**) Samples from high-brightness, complex noise scenes. (**Right**) Horizontally flipped mirror-image augmented samples. (Note: White circles and red dots mark observation-sensitive zones, not detection targets.)

To ensure evaluation fairness, the 2000 raw images were divided into training, validation, and test sets in an 8:1:1 ratio. Subsequently, to enhance model generalization, data augmentation was applied exclusively during training by implementing geometric transformations such as rotation and mirroring on training-set images, as shown in Figure 1.

### 2.2. Characterization of the Dataset

**Figure 2:**
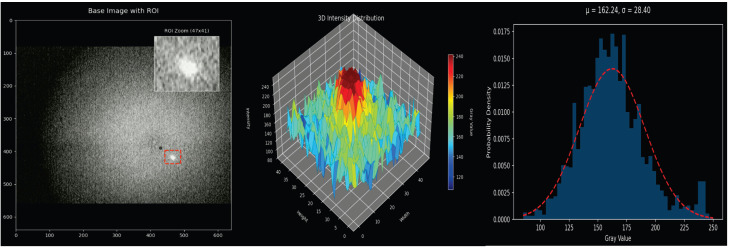
Target analysis.

To further parse the target’s imaging characteristics, this study conducts a grayscale modeling analysis on typical samples (Figure 2, Right). The locally magnified region in Figure 2 (Left) reveals that SLR targets possess characteristics such as low signal-to-noise ratio (SNR), strong background noise interference, and blurred edges. The 3D grayscale distribution (Figure 2, Middle) indicates that the target’s energy is highly concentrated in the central region and rapidly decays outward in a radial pattern, exhibiting characteristics of an approximate Gaussian distribution.

Theoretically, this Gaussian morphology is not accidental but stems from the physical propagation properties of the laser beam. Due to atmospheric turbulence (commonly referred to as astronomical “seeing”) and the diffraction limits of the telescope’s optical system, the laser return signal—conceptually a point source—spreads into a diffuse spot. This phenomenon is mathematically described by the Point Spread Function (PSF), which, in ground-based SLR observations, is accurately modeled by a 2D Gaussian distribution. Furthermore, while system errors such as optical axis deviations or ephemeris errors may shift the target’s centroid position on the CCD, they do not alter this fundamental intensity distribution profile. This physical reality serves as the theoretical foundation for our network design: standard rectangular convolution kernels (which sample uniformly) are suboptimal for such targets, whereas a Gaussian-matching strategy aligns intrinsically with the signal’s energy decay.

This property poses a dual challenge, requiring the detection algorithm to have both high central sensitivity and robust noise suppression capabilities at the edges.

Through the aforementioned dataset construction and target feature analysis, this work not only provides a solid data foundation for the design of the subsequent methods but also reveals the severe challenges faced by small object detection in SLR CCD imagery, thereby offering a theoretical basis for the proposal of innovative detection algorithms.

## 3. Method

### 3.1. GMF-Net Overall Architecture

**Figure 3:**
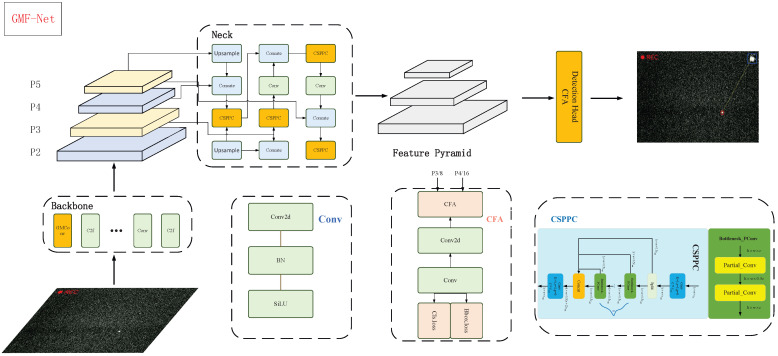
The overall architecture of GMF-Net.

**Figure 4:**
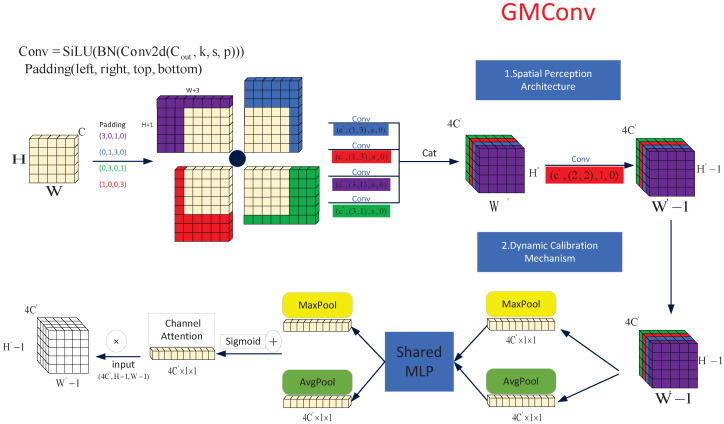
GMConv.

**Figure 5:**
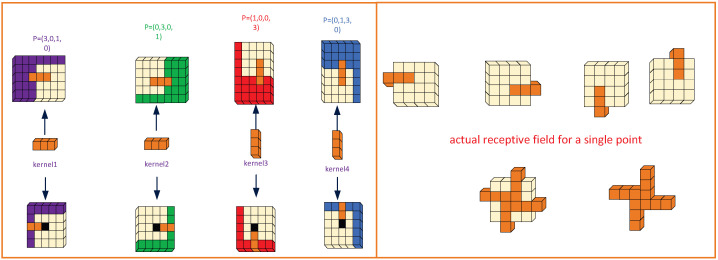
Receptive Field.

**Figure 6:**
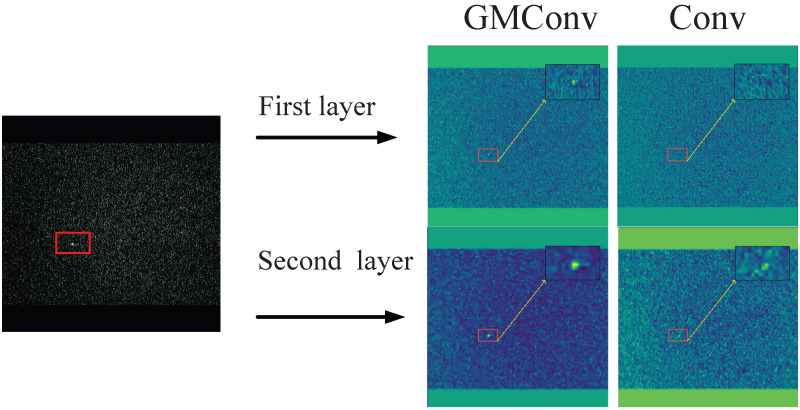
Convolutional Visualization.

To address the challenges of detecting extremely small targets with low signal-to-noise ratios and complex backgrounds in SLR system CCD images, this study proposes a Gaussian-Matched Fusion Network (GMF-Net) (see Figure 3, Figure 4, Figure 5 and Figure 6). The overall architecture draws inspiration from the efficient single-stage detection pipeline of the YOLO series but has been systematically redesigned to accommodate the unique characteristics of SLR imagery. GMF-Net comprises three key modules, each specifically optimized for the SLR context.

- **Gaussian-Matched Convolution (GMConv):** The GMConv module is designed based on the Gaussian energy distribution characteristics analyzed in Section 2.2. Unlike conventional convolutions with uniform receptive fields, GMConv employs a synergistic mechanism of multi-directional heterogeneous sampling and dynamic channel calibration. This design precisely models the spatial energy decay of the target (i.e., dense center and sparse periphery), thereby adaptively enhancing target responses across channels while effectively suppressing background noise.
- **Cross-Stage Partial Pyramidal Convolutional Network (CSPPC):** The CSPPC module constructs the feature fusion neck of the network. It first decouples input features into modeling and information streams by channel, then expands multi-scale receptive fields through pyramid-cascaded partial convolutions (Partial Conv). This architecture achieves a 30% reduction in parameters and a 25% decrease in GFLOPs, maintaining competitive detection accuracy with significantly improved computational efficiency.
- **Cross-Feature Attention (CFA):** To enhance fusion efficiency in the multi-scale detection head, the CFA module dynamically integrates shallow high-resolution features (P3) with deep semantic features (P4) using a local attention mask. This mechanism establishes an adaptive balance between fine-grained spatial details and global contextual semantics, resulting in more precise bounding box regression and improved robustness in small-target detection.

GMF-Net achieves multidimensional feature enhancement and recognition of minute targets while improving computational efficiency through the synergistic optimization of Gaussian Matching Convolution, Partial Pyramid Convolution, and Cross-Layer Attention.

### 3.2. Gaussian Matching Convolution (GMConv)

The Gaussian distribution characteristic of SLR-CCD targets (Section 2.2) cannot be accurately matched by the symmetric, uniform sensing approach of traditional rectangular convolution kernels. The Gaussian Matching Convolution (GMConv) proposed herein achieves precise matching of target energy characteristics through a synergistic mechanism combining multi-directional feature enhancement and dynamic channel calibration (as shown in Figure 4). This mechanism enables central dense sampling, radial sparse sampling, and adaptive weight adjustment. Specifically, GMConv applies four-directional asymmetric padding $F_{{\mathrm{in}}_{P(L,R,T,B)}}^{C\times H\times W}$ to the input feature map $F_{\mathrm{in}}^{C\times H\times W}$ before convolving it with heterogeneous directional convolutional kernels ${\left\{K_{i}\right\}}_{i=1}^{4}$. Following the convolution operation, batch normalization (as shown in Equations (1)–(4)) and channel concatenation (as shown in Equation (5)) are performed. Ultimately, convolutional kernels are utilized to adjust the output dimension, thereby achieving compatible interchangeability with standard convolutional layers. This structure not only implements both centrally dense sampling and diagonally radial sparse sampling, but also extends the receptive field from the original 3×3 to 3×4, significantly enhancing its ability to perceive targets (Figure 5).

(1) $$\begin{array}{ccccc} & & F_{1}^{C^{\prime }\times H^{\prime }\times W^{\prime }}=\mathrm{SiLU}\left(\mathrm{BN}\left(F_{{\mathrm{in}}_{P(3,0,1,0)}}^{C\times H\times W}⊗K_{1}^{C^{\prime }\times 1\times 3}\right)\right) & & \end{array}$$

(2) $$\begin{array}{ccccc} & & F_{2}^{C^{\prime }\times H^{\prime }\times W^{\prime }}=\mathrm{SiLU}\left(\mathrm{BN}\left(F_{{\mathrm{in}}_{P(0,1,3,0)}}^{C\times H\times W}⊗K_{2}^{C^{\prime }\times 3\times 1}\right)\right) & & \end{array}$$

(3) $$\begin{array}{ccccc} & & F_{3}^{C^{\prime }\times H^{\prime }\times W^{\prime }}=\mathrm{SiLU}\left(\mathrm{BN}\left(F_{{\mathrm{in}}_{P(0,3,0,1)}}^{C\times H\times W}⊗K_{3}^{C^{\prime }\times 1\times 3}\right)\right) & & \end{array}$$

(4) $$\begin{array}{ccccc} & & F_{4}^{C^{\prime }\times H^{\prime }\times W^{\prime }}=\mathrm{SiLU}\left(\mathrm{BN}\left(F_{{\mathrm{in}}_{P(1,0,0,3)}}^{C\times H\times W}⊗K_{4}^{C^{\prime }\times 3\times 1}\right)\right) & & \end{array}$$

(5) $$\begin{array}{cc} F_{\mathrm{cat}}^{4C^{\prime }\times H^{\prime }\times W^{\prime }}= & \mathrm{Cat}(F_{1}^{C^{\prime }\times H^{\prime }\times W^{\prime }},\\ \; & F_{2}^{C^{\prime }\times H^{\prime }\times W^{\prime }},\\ \; & F_{3}^{C^{\prime }\times H^{\prime }\times W^{\prime }},\\ \; & F_{4}^{C^{\prime }\times H^{\prime }\times W^{\prime }}) \end{array}$$

where ⊗ is the convolution operator and P(L,R,T,B) represents pixel filling in the four directions (left, right, up, and down). To ensure strict spatial alignment for concatenation, the convolution stride (*s*) is set to 1 for all branches. Additionally, the bias term is omitted (bias=False) since the linear scaling is handled effectively by the subsequent Batch Normalization (BN) layer.

Subsequently, the multi-directional feature-enhanced features are processed by global max pooling and average pooling, respectively, to extract dual-path complementary features (Formulas (6) and (7)). A multi-layer perceptron with shared weights is then utilized to non-linearly map the extracted features (Formulas (8) and (9)) and generate a channel weight matrix $A_{c}$, which adaptively quantifies the contribution of each channel to target detection and suppresses background noise channels (Formula (10)). Finally, multiplicative broadcasting is employed for feature calibration (Formula (11)).

(6) $$\begin{array}{c} F_{\text{max}}=\text{MaxPool}\left(F_{\mathrm{cat}}\right)\in R^{4C^{\prime }\times 1\times 1} \end{array}$$

(7) $$\begin{array}{c} F_{avg}=\text{AvgPool}\left(F_{\mathrm{cat}}\right)\in R^{4C^{\prime }\times 1\times 1} \end{array}$$

(8) $$\begin{array}{cc} F_{max}^{\prime }=W_{2}\cdot \delta & \left(W_{1}\cdot F_{max}\right)(W_{1}\in R^{4C^{\prime }\times 4C^{\prime }},\\ \; & W_{2}\in R^{4C^{\prime }\times 4C^{\prime }}) \end{array}$$

(9) $$\begin{array}{c} F_{avg}^{\prime }=W_{2}\cdot \delta \left(W_{1}\cdot F_{avg}\right) \end{array}$$

(10) $$\begin{array}{c} A_{c}=\sigma \left(F_{max}^{\prime }+F_{avg}^{\prime }\right)\in R^{4C^{\prime }\times 1\times 1} \end{array}$$

(11) $$\begin{array}{c} F_{out}=A_{c}⊗F_{\mathrm{cat}}\in R^{H_{2}\times W_{2}\times C_{2}} \end{array}$$

To clarify the theoretical novelty, we further compare GMConv with existing irregular convolution operators. Unlike Deformable Convolution [30], which learns offsets in a purely data-driven manner but lacks specific structural constraints, and Pinwheel Convolution [31], which is tailored for strip-like thermal signatures, GMConv is explicitly grounded in the physical Point Spread Function (PSF) of SLR optical systems. By restricting the sampling pattern to a fixed “central-dense, radial-sparse” Gaussian topology, GMConv introduces a strong physical prior. This design is significantly more data-efficient and robust for star-like spot detection than generic deformable kernels, which may struggle to converge or overfit to background noise when processing such tiny, symmetric targets under low SNR conditions.

In essence, GMConv accurately captures energy decay features in the spatial dimension while dynamically adjusting feature weights in the channel dimension. This capability enables the fused feature map to both conform to the true energy distribution of the target and possess interference resistance against lighting variations and complex noise. This establishes a highly discriminative feature representation system for small object detection tasks.

#### GMConv Feature Map Visualization

To illustrate the merits of GMConv in comparison to conventional convolution, feature maps were extracted from the primary trunk for the purpose of visual analysis (see Figure 6).

As demonstrated by the convolutional visualization, GMConv has been shown to be capable of extracting clearer edge information even in the initial convolutional output. In the second layer’s feature map, target contours are more prominent, and background noise response is significantly reduced. A comparison of GMConv with traditional convolution reveals its superior performance in preserving spatial structures and enhancing details. This finding validates the robustness and feature extraction capabilities of GMConv in low signal-to-noise ratio scenarios.

### 3.3. Lightweight Neck Network: CSPPC

**Figure 7:**
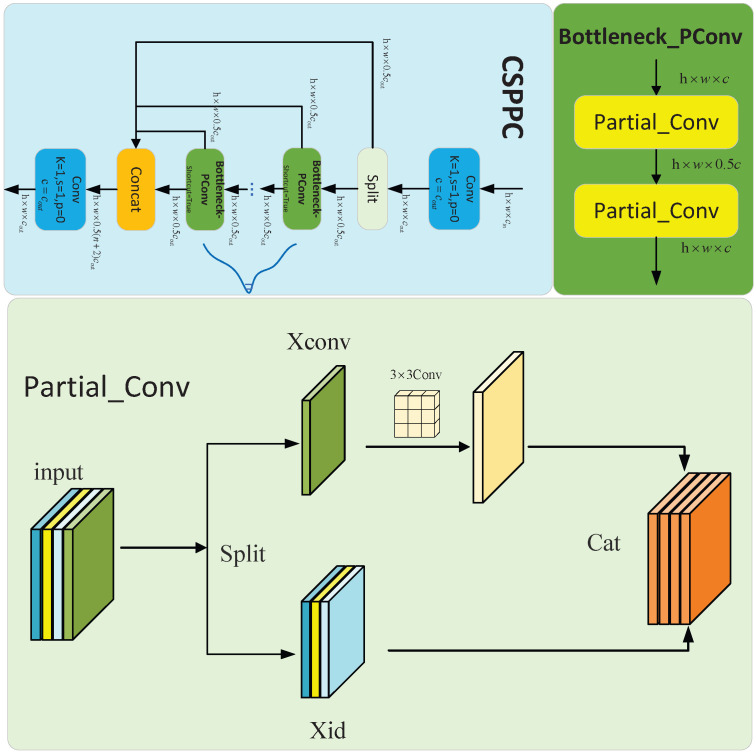
CSPPC structure.

In traditional Cross-Stage Partial (CSP) networks, the C2f module uniformly applies convolution operations across all channels of the input features, failing to fully leverage the redundancy among feature channels. This design exhibits particular inefficiency during the processing of high-resolution feature maps, resulting in a substantial increase in FLOPs. This, in turn, imposes severe constraints on the deployment of models on platforms with limited computing capabilities. Inspired by FasterNet [6], this paper introduces a partial convolution mechanism into the CSP architecture, replacing the bottleneck structure. By applying convolution to only a subset of feature map channels, redundant information within the feature maps is fully exploited, computation and memory access overlap is reduced, and the computational cost is effectively lowered (as illustrated in the Figure 7).

#### CSPPC Improvement Comparison

Comparative experiments were conducted to assess the impact of the CSPPC partial convolution mechanism. The results demonstrate that the CSPPC module enhances detection accuracy while significantly improving efficiency, reducing the number of parameters by 30% and GFLOPs by 25% compared to the conventional CSP structure. This outcome indicates that CSPPC exhibits both exceptional accuracy and lightweight capabilities.

### 3.4. Cross-Feature Fusion Attention CFA

In the context of SLR-CCD images, the identification of small targets presents a particular challenge due to the diminution of target features. Deep-layer downsampling operations frequently result in the dilution of semantic information concerning minute objects. Conversely, shallow layers, while preserving details, exhibit a paucity of semantic support. Concurrently, the independent prediction of multi-scale branches serves to sever the association between local details and global context.

**Figure 8:**
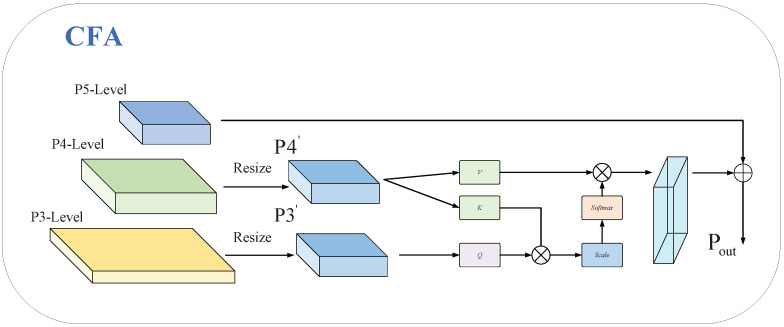
CFA structure.

In order to address these issues, the present paper proposes a CFA cross-feature fusion attention mechanism, which is then embedded into the detection head. The implementation of attention-based fusion in the P3 and P4 detection layers has been demonstrated to facilitate multi-scale collaborative perception, thereby enhancing the accuracy and robustness of detection for small objects. The overall structure is illustrated in the Figure 8.

The first step in the CFA process involves the unification of the spatial dimensions of the P3 and P4 layers with the P5 layer. Subsequently, the unified-dimension P3 and P4 layers are projected into Q, K, and V spaces (Formula (12)). Finally, cross-layer features are integrated based on attention weights, and a residual connection is incorporated to preserve the original deep-layer feature information flow (Formula (13)).

(12) $$Q=P_{3}^{\prime }W^{Q},K=P_{4}^{\prime }W^{K},V=P_{4}^{\prime }W^{V}$$

(13) $$F_{M}=P_{5}^{\prime }+\mathrm{softmax}\left(\frac{\left(P_{3}^{\prime }W^{Q}\right){\left(P_{4}^{\prime }W^{K}\right)}^{T}}{\sqrt{d_{k}}}\right)\left(P_{4}^{\prime }W^{V}\right)$$

The design outlined above establishes a dynamic attention bridge between shallow and deep features. This approach not only preserves high-resolution details but also incorporates deep semantic guidance, enabling the network to adaptively focus on tiny target regions and effectively suppress complex background noise. Experimental results confirm the effectiveness of this enhancement, showing increases of 3.5% in mAP50 and 2.5% in mAP50–95. These gains highlight the CFA detection head’s robust target recognition capability in complex environments.

## 4. Experiment

### 4.1. Experimental Environment and Implementation Details

The configuration of the experimental environment is shown in Table 1.

The training parameters are set as follows: the training period is 100 epochs, the batch size is set to 32, and the image size is set to 640×640. The model parameters are optimized using the SGD optimizer, with the initial learning rate set to 0.01 and the momentum parameter set to 0.937. To ensure a fair and unbiased comparison with baseline models, we strictly adopted these default hyperparameter settings from the official YOLO repository, avoiding specific parameter tuning that might favor our proposed method.

To prevent overfitting, the model uses a weight decay strategy, with the weight decay value set to $5\times 10^{-4}$. Furthermore, we implemented an early stopping mechanism (with patience set to 50 epochs) based on the validation loss to automatically terminate training when convergence was reached. Standard data augmentation techniques, including Mosaic and Mixup, were also applied during the training phase to introduce data diversity and enhance the model’s generalization capability on unseen test data.

### 4.2. Experimental Evaluation Criteria

In order to comprehensively evaluate the effectiveness of the model, this paper selects precision (P), recall (R), mAP50, and mAP50–95 as indicators (Equations (14) to (16)). In addition, this paper also uses Params and GFLOPS to compare the parameters and running speed of the model.

(14) $$\begin{array}{cccc} & & \mathrm{Precision}=\frac{\mathrm{TP}}{\mathrm{TP}+\mathrm{FP}} & \end{array}$$

(15) $$\begin{array}{cccc} & & \mathrm{Recall}=\frac{\mathrm{TP}}{\mathrm{TP}+\mathrm{FN}} & \end{array}$$

(16) $$\begin{array}{cccc} & & \mathrm{mAP}=\frac{1}{N}\sum_{i=1}^{N}AP_{i} & \end{array}$$

where **TP** (True Positive) denotes the number of correctly predicted true cases, **FP** (False Positive) denotes the number of incorrectly predicted true cases, and **FN** (False Negative) denotes the number of incorrectly predicted non-true cases. **AP** stands for the Average Precision of a single category; N denotes the total number of categories.

### 4.3. Comparison Test—Comparing Different Backbone Network

This paper conducts a comparison of the detection performance of diverse backbone networks (such as CSPDarknet53, the EfficientViT series, Fastnet, Ghostnet, the MobileNet series, the ShuffleNet series, and the GMConv proposed herein) within the YOLOv8 framework. To ensure the fairness of the comparison, all models employed in this experiment shared the exact same Neck and Detection Head architectures, with the backbone network being the only variable. Furthermore, all models were trained using identical hyperparameter settings (e.g., optimizer, learning rate, and batch size) as detailed in Section 4.1. The comparison outcomes on the test set are presented in Table 2.

#### Comprehensive Performance Analysis

As shown in the comparative results in Table 2, the core advantage of GMConv as a backbone network lies in its detection accuracy, achieving optimal performance across multiple metrics:

- **Optimal Global Detection Accuracy:** GMConv achieves the highest precision (P = 90.0%) and recall (R = 85.3%), representing improvements of 4.8 and 6.5 percentage points, respectively, over the baseline CSPDarknet53 (P = 85.2%, R = 78.8%). Concurrently, its mAP@50 reaches 91.8%, which is 7.8 percentage points higher than CSPDarknet53 and significantly superior to PConv (89.9%) and ShuffleNetV1 (89.1%), demonstrating the strongest global detection capability.
- **Accuracy–Efficiency Trade-off:** In the trade-off between parameters and computational cost, GMConv demonstrates outstanding efficiency. Its parameter count is 3.0 M, which is on par with the baseline model and PConv. In terms of computation, its GFLOPs are 4.3 G, only a minor increase compared to the baseline (4.0 G) and PConv (4.2 G). This minimal computational overhead (+0.3 GFLOPS) is exchanged for a substantial +7.8% increase in mAP@50, proving that the module is extremely efficient.

In summary, GMConv, by precisely matching the Gaussian energy distribution, significantly enhances the detection accuracy and recall of small targets at a minimal computational cost, proving its superiority in the context of small object detection in SLR imagery.

### 4.4. Ablation Experiments

**Figure 9:**
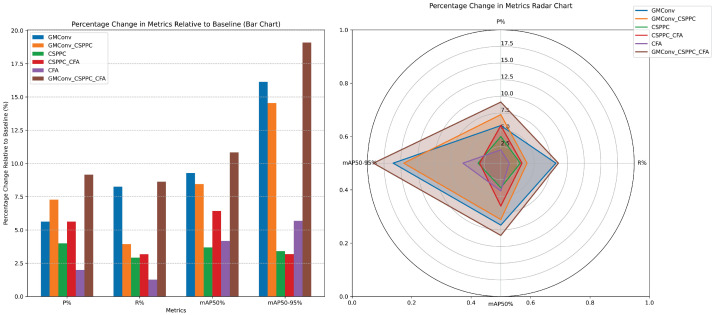
Comparison of ablation experiments.

To verify the independent contributions and synergistic effects of each module on detection performance, we conducted detailed ablation experiments on different module combinations across multiple metrics, including accuracy, recall, mAP, number of parameters (Params/M), and computational complexity (GFLOPS). Table 3 summarizes the configurations of each experimental group and their corresponding performance indicators, while Figure 9 presents the ablation experiment results as bar charts and radar charts.

#### Ablation Experiments Conclusion

1. **Single-module Performance**
  - **GMConv (Group 2 vs. 1)**: **GMConv** yields the most significant gains, with precision up by 4.8%, recall up by 6.5%, mAP50 up by 7.8%, and mAP50–95 up by 7.1%, while only increasing computation by 0.3 GFLOPS.
  - **CSPPC (Group 3 vs. 1)**: Achieves lightweight design alongside performance improvement: Precision +3.4%, recall +2.3%, mAP50 +3.1%, mAP50–95 +1.5%. Model size reduced to 2.1 M parameters and 3.0 GFLOPS.
  - **CFA (Group 4 vs. 1)**: **Improves accuracy**, with precision +1.7%, recall +1.0%, mAP50 +3.5%, mAP50–95 +2.5%; GFLOPS slightly reduced to 3.8 **GFLOPS**.
2. **Multi-module Collaboration**
  - **Two-module Combinations**
    - GMConv + CSPPC (Group 6): Maintains a lightweight footprint (2.2 M parameters) while further boosting mAP50 to 91.1% and mAP50–95 to 50.4%, enhancing small object detection.
    - CSPPC + CFA (Group 5): Although the smallest model (2.2 M parameters), it shows a slight drop in recall and mAP@50–95, highlighting the critical role of GMConv for fine-feature extraction.
  - **Three-module Fusion**: Integrating GMConv, CSPPC, and CFA (Group 7) achieves the best balance: precision 93.0%, recall 85.6%, mAP50 93.1%, mAP50–95 52.4%; only 2.2 M parameters and 2.9 GFLOPS. Compared to baseline YOLOv8, it substantially improves detection accuracy while reducing both computation and model size.
3. **Overall Conclusions**
  - GMConv alone provides the largest boost, significantly enhancing small object detection capability.
  - CSPPC and CFA each contribute significantly to lightweight design and detection capabilities.
  - The combination of all three modules delivers the highest detection accuracy and recall, with an extremely compact model and excellent real-time performance, validating the overall effectiveness of the GMF-Net design.

### 4.5. Comparison Experiments of Different Models

GMF-Net performs best on a number of key indicators, as shown in the comparative results in Table 4.

1. **Optimal Detection Accuracy and Robustness**
  - **GMF-Net**: GMF-Net ranks first in all key metrics: precision P reaches 93.0%, mAP50 reaches 93.1%, with both being the highest values; recall R is 85.6%, on par with YOLOv9 and far superior to other detectors. This balance is critical for practical SLR applications. High recall is essential to prevent the loss of valuable satellite passes (avoiding False Negatives), while high precision is a requisite to minimizing false tracking commands triggered by background noise (avoiding False Positives). Unlike baseline models that exhibit trade-offs—such as YOLOv5 (High P, Moderate R) which risks data loss, or YOLOv9 (High R, Lower P) which introduces noise—GMF-Net achieves the optimal equilibrium, ensuring both data completeness and tracking efficiency.
  - **Transformer-Based RT-DETR**: RT-DETR has inherent advantages in context modeling, its mAP50 is below 87% due to extremely small target sizes and complex background noise in CCD images, limiting its detection performance.
  - **Two-Stage Methods (Faster R-CNN, SSD300)**: Performs poorly in SLR small object detection, demonstrating limited adaptability to SLR small object detection tasks.
2. **Lightweight model and easy deployment**
  - **GMF-Net**: Achieves optimal accuracy with only 2.2 million parameters and 2.9 GFLOPs of computational complexity, outperforming mainstream lightweight detectors such as YOLOv9/v10/v11.
  - **RT-DETR**: Requires over 100M parameters and hundreds of GFLOPs of computational overhead, making it highly unsuitable for deployment on edge devices. Our model demonstrates a clear advantage in resource-constrained scenarios.

GMF-Net achieves the triple optimal performance of ‘high accuracy, high efficiency, and strong adaptability’ in small object detection tasks. It not only significantly outperforms current mainstream detectors but is also, thanks to its lightweight structure, particularly suitable for Satellite Laser Ranging (SLR) processing scenarios with strict requirements for real-time performance and resource consumption, demonstrating excellent engineering application prospects.

### 4.6. Statistical Significance and Reproducibility Analysis

To ensure the reliability and reproducibility of our experimental results, we conducted a rigorous statistical analysis. Specifically, we performed five independent runs for both the baseline (YOLOv8) and our proposed GMF-Net using different random seeds (Seed 0 to 4). The quantitative results, reported as Mean ± Standard Deviation (SD), are summarized in Table 5.

As presented in the table, GMF-Net demonstrates consistent performance improvements across all evaluation metrics. In terms of detection accuracy, our method increases the mean mAP50 by 9.3% (0.835→0.928) and mAP50–95 by 8.5% (0.438→0.523). Notably, regarding the Precision metric, GMF-Net not only improves the mean value but also reduces the standard deviation from 0.014 to 0.012, indicating that our module contributes to better stability against random initializations.

To further validate that these improvements are statistically significant and not the result of stochastic fluctuations, we performed independent two-sample Student’s *t*-tests. We calculated the *t*-values and *p*-values for each metric. As shown in Table 5, the *t*-statistics for all metrics are exceedingly high (e.g., t=17.20 for mAP50 and t=13.76 for recall), yielding *p*-values significantly below 0.001. These statistical evidences strongly confirm the robustness and effectiveness of the proposed GMF-Net.

### 4.7. Visualization Experiments

To more intuitively demonstrate the detection performance of the proposed GMF-Net model, we conduct visualization comparison experiments from three perspectives: (1) detection result visualization, (2) heatmap response analysis, and (3) confusion matrix evaluation.

#### 4.7.1. Detection Result Visualization

**Figure 10:**
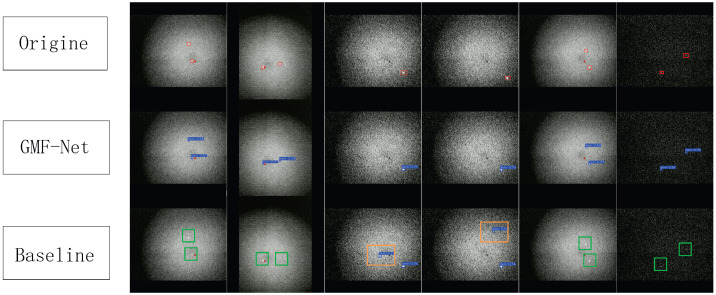
Detection Result Visualization. (Note: This visualization uses a custom color scheme differing from YOLO defaults: Blue = TP, Orange = FP, Green = FN).

Several representative satellite CCD image samples are selected to compare GMF-Net with the baseline YOLOv8. As shown in Figure 10, the first row shows the manually annotated ground truth, the second row shows the GMF-Net detections, and the third row shows the YOLOv8 detections. We adopted a custom color scheme distinct from standard YOLO defaults: Blue boxes indicate correct detections, red boxes indicate ground truth targets, and orange/green boxes represent False Positives and False Negatives, respectively. The comparison shows that YOLOv8 produces two False Positives and eight False Negatives, while GMF-Net produces none, demonstrating superior detection accuracy and robustness.

#### 4.7.2. Failure Case Analysis

To provide deeper insights into the robustness of the proposed method, we analyzed the specific causes of the detection errors (FPs and FNs) observed in the baseline model:

- **False Positives (FPs):** In the baseline YOLOv8 results, FPs primarily originate from high-intensity background noise clusters (e.g., star clutter or hot pixels) that structurally mimic the intensity peaks of small targets. The standard convolution kernels struggle to differentiate these noise artifacts from valid Gaussian targets. In contrast, GMF-Net effectively suppresses these FPs by utilizing the GMConv module, which is physically grounded in the Gaussian energy distribution, allowing the network to filter out irregular noise patterns that do not match the target’s physical morphology.
- **False Negatives (FNs):** FNs predominantly occur when targets exhibit extremely low signal-to-noise ratios (SNRs), causing them to blend into the background readout noise. The baseline model fails to extract these faint features due to insufficient feature enhancement. GMF-Net mitigates this through the synergistic effect of GMConv (which enhances central feature response) and the CFA module (which fuses multi-scale semantic information), thereby significantly improving the recall rate for ultra-weak targets.

#### 4.7.3. Heatmap Response Analysis

**Figure 11:**
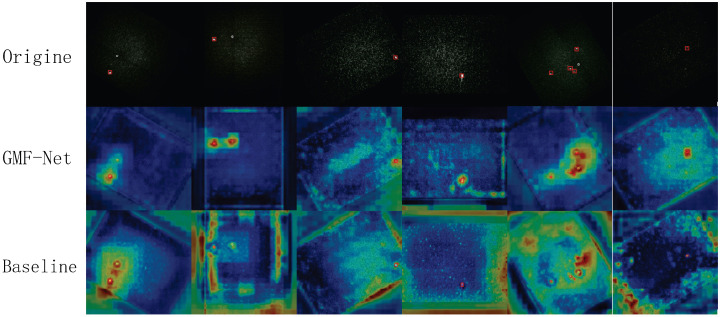
Heatmap.

To further investigate each model’s attention to faint target features, we generate heatmaps for the same samples, including the original annotations, GMF-Net heatmaps, and YOLOv8 heatmaps (see Figure 11). The comparison shows remarkable differences:

- GMF-Net produces prominent activations only at true target locations with a high signal-to-noise ratio.
- YOLOv8 shows general "heating" with less distinct target peaks, often responding to background textures.
- For extremely weak targets, GMF-Net remains stable and concentrated, while YOLOv8 gives almost no response or only scattered weak signals.
- For clustered targets, GMF-Net’s hotspots align closely with true positions, while YOLOv8 generates multiple false alarms in background regions.
- For low-contrast isolated points, GMF-Net maintains concentrated peaks (albeit weaker ones), indicating greater robustness; YOLOv8’s responses are scattered and prone to misses.

These observations suggest that GMF-Net’s improvements in noise suppression and feature modeling significantly enhance activation concentration and prominence for extremely faint targets, while effectively suppressing background noise. In contrast, YOLOv8’s heatmap responses are scattered and suffer from false highlights and missed detections, hindering accurate localization and subsequent tracking.

#### 4.7.4. Generalization Experiment

To further validate applicability in other faint-target scenarios, we evaluated both models on the IRSTD-1k infrared small-target dataset, which shares similar characteristics with SLR-CCD images. Results are presented in Table 6. On IRSTD-1k [42], GMF-Net improves precision and recall by 3.8% and 1.8%, respectively, and achieves a 2.3-point gain in mAP50–95. These findings further confirm the strong generalization and adaptability of the proposed method across diverse small-target detection tasks.

## 5. Conclusions

This paper addresses the challenges of tiny targets and low signal-to-noise ratios in Satellite Laser Ranging (SLR) small object detection by proposing a Gaussian-Matched Fusion Network (GMF-Net). This network is inspired by the efficient single-stage pipeline architecture of the YOLO series but achieves a three-stage synergistic optimization tailored to the unique physical properties of the SLR scenario, such as the Gaussian energy distribution discussed in (Section 2.2).

First, targeting the approximate Gaussian energy distribution characteristic of SLR targets, this paper designs Gaussian-Matched Convolution (GMConv) to replace traditional convolutions in the backbone. This module employs a mechanism of four-directional asymmetric sampling and dynamic channel calibration, significantly enhancing the model’s capability to capture small object features. Second, a lightweight Cross-Stage Partial Pyramidal Convolution (CSPPC) is integrated, drastically reducing parameters and computational load while maintaining accuracy. Finally, the designed Cross-Feature Attention (CFA) module effectively fuses shallow-layer details with deep-layer semantic features. Combined with residual calibration and a DFL decoder, it adaptively focuses on tiny target regions, improving detection accuracy and robustness.

Experiments on our self-built SLR dataset demonstrate that, compared to baseline models and mainstream algorithms, GMF-Net not only achieves higher accuracy but also consumes fewer resources. It strikes a superior balance between precision and performance, providing a feasible and efficient solution for small object detection in resource-constrained environments.

In the future, we will further optimize model efficiency by compressing computational redundancy through model pruning techniques. Concurrently, we will introduce a temporal modeling module to incorporate motion consistency features across consecutive CCD frames, building a dynamic detection strategy to address the trajectory drift problem of high-speed moving targets. These improvements will drive the intelligent upgrading of SLR systems in low-signal, high-noise scenarios, providing critical technical support for deep space exploration and spatial object monitoring.

## Figures and Tables

**Table 1:** Experimental Environment Configuration.

Operating System	Ubuntu 22.04
GPU	RTX 4090 (24 GB)
CPU	16 VCPU Intel(R) Xeon(R) Platinum 8352V CPU @ 2.10 GHz
Memory	120 GB
Programming Languages	Python 3.10
Frameworks	PyTorch 2.1.0 + CUDA 12.1
IDE	JupyterLab

**Table 2:** Performance comparison of different backbone networks within the YOLOv8 Framework. Best results are shown in **bold**, and second-best are underlined.

Backbone Network	P%	R%	mAP50%	mAP50–95%	Param (M)	GFLOPS (G)
CSPDarknet53 (baseline)	85.2	78.8	84.0	44.0	**3.0**	4.0
Fastnet [32]	88.7	84.9	91.1	51.4	15.2	18.5
Ghostnet [33]	86.2	80.3	85.8	42.2	6.3	4.3
MobileNetV1 [34]	87.3	84.3	89.9	**52.4**	6.1	7.8
Pconv [31]	**88.9**	84.0	89.9	49.2	**3.0**	4.2
shufflenet_v1 [35]	88.5	82.1	89.1	50.6	3.5	**3.2**
Starnet [36]	87.2	81.3	87.5	48.1	**3.0**	8.3
Ours (GMConv)	**90.0**	**85.3**	**91.8**	51.1	**3.0**	4.3

**Table 3:** Ablation experiments of different module combinations. Best results are shown in **bold**, and second-best are underlined.

Group	GMConv	CSPPC	CFA	P%	R%	mAP50%	mAP50–95%	Params (M)	GFLOPS (G)
1				85.2	78.8	84.0	44.0	3.0	4.0
2	✓			90.0	85.3	91.8	51.1	3.0	4.3
3		✓		88.6	81.1	87.1	45.5	**2.1**	3.0
4			✓	86.9	79.8	87.5	46.5	3.0	3.8
5		✓	✓	90.0	81.3	89.4	45.4	2.2	**2.7**
6	✓	✓		91.4	81.9	91.1	50.4	2.2	3.2
7	✓	✓	✓	**93.0**	**85.6**	**93.1**	**52.4**	2.2	2.9

**Table 4:** Performance comparison with state-of-the-art object detection models. Best results are shown in **bold**, and second-best are underlined.

Mode	P%	R%	mAP50%	mAP50–95%	Params (M)	GFLOPS/G
YOLOv5	91.9	83.4	90.2	**53.0**	2.5	3.5
YOLOv6 [37]	86.8	78.9	84.2	44.0	4.2	5.9
YOLOv9 [38]	90.9	**85.6**	91.1	52.4	**2.0**	3.8
YOLOv10 [24]	88.1	81.2	89.1	51.7	2.7	4.1
YOLOv11 [39]	88.7	81.4	89.7	48.9	2.6	3.2
YOLOv12 [40]	88.6	78.1	84.9	42.8	2.6	3.2
Faster RCNN [17]	29.4	6.3	2.96	0.1	137.1	370.2
SSD300(Vgg) [18]	0	0	0	0	26.2	62.7
RT-DETR-resnet101 [41]	88.4	82.4	86.4	39.1	60.9	186.2
RT-DETR-l [41]	83.2	79.6	81.8	40.9	63.1	103.4
Ours (GMF-Net)	**93.0**	**85.6**	**93.1**	52.4	2.2	**2.9**

**Table 5:** **Statistical Significance Analysis.** Comparison between YOLOv8 and GMF-Net (N=5). We report the Mean Difference (Δ) and the *t*-statistic computed via Student’s *t*-test. All improvements are statistically significant (p<0.001).

Metric	YOLOv8	GMF-Net (Ours)	Δ Mean	*t*-Value
mAP50	0.835±0.005	0.928±0.011 *	(+0.093)	17.20
mAP50–95	0.438±0.007	0.523±0.027 *	(+0.085)	06.81
Precision	0.844±0.014	0.919±0.012 *	(+0.075)	09.12
Recall	0.791±0.004	0.863±0.011 *	(+0.072)	13.76

* indicates p<0.001. Degrees of freedom df≈8.

**Table 6:** Evaluation on IRSTD-1k infrared small-target dataset. Best results are shown in **bold**, and second-best are underlined.

Model	P%	R%	mAP50%	mAP50–95%
YOLOv8 (base)	89.6	75.8	84.7	39.8
GMF-Net	**93.4**	**77.6**	**85.3**	**42.1**

## Data Availability

The data presented in this study are available on request from the corresponding author. The data are not publicly available due to the sensitive nature of satellite observation data and institutional privacy policies.
